# Structural dataset for Si(1 1 0) and Si(17 15 1) surface models and related calculated STM images

**DOI:** 10.1016/j.dib.2019.104847

**Published:** 2019-11-26

**Authors:** Ruslan Zhachuk

**Affiliations:** A.V. Rzhanov Institute of Semiconductor Physics, SB RAS, Novosibirsk 630090, Russia

**Keywords:** STM, DFT, Si(110), Ge(110), Surface, Reconstruction, Pentamer, Modelling

## Abstract

In this work we present the novel atomic models of the (1 1 0)-(16 × 2), (1 1 0)-*c*(8 × 10), (1 1 0)-(5 × 8) and (17 15 1)-(2 × 1) silicon surface reconstructions. The models are also valid for respective germanium surfaces. The dataset reports atomic coordinates for each surface reconstruction and related calculated bias-dependent scanning tunneling microscopy (STM) images. The data were obtained using the standard first-principles density functional theory calculations. The atomic models reported in this dataset are based on the universal building block for (1 1 0)-family silicon and germanium surfaces, proposed by R.A. Zhachuk and A.A. Shklyaev [1] and a vast number of STM data published in the literature. For comparison the data for the Si(1 1 0)-(16 × 2) older models by Stekolnikov et al. [2] and Yamasaki et al. [3] are also given. The presented models and related calculated scanning tunneling microscopy images allow to derive experimentally testable hypotheses and to interpret the experimental data. The reported atomic coordinates can be directly reused in other calculations related to Si(1 1 0) and Ge(1 1 0) surfaces provided that this work is cited.

**Table:** Specifications Table

Subject	Surfaces and Interfaces
Specific subject area	Atomistic first principles calculation
Type of data	Structural Data: atomic model coordinates in XYZ format and respective translation vectors. Figures: atomic models and related calculated STM images
How data were acquired	The data were acquired by means of first-principles density functional theory calculations. The calculations were carried out using the pseudopotential [4] density functional theory SIESTA code [5] (version 4.1) within the local density approximation to the exchange and correlation interactions between electrons [6]. The valence states were expressed as linear combinations of the Sankey-Niklewski-type numerical atomic orbitals [5]. The constant-current STM images were produced based on the Tersoff-Hamann approximation [7] using the eigenvalues and eigenfunctions of the Kohn-Sham equation [8] for a relaxed atomic structure. The WSxM software was used to process the calculated STM images [9].
Data format	Raw
Parameters for data collection	Standard bulk-optimized DZP functions [5] were assigned for all species for relaxing the structure. The surface-optimized DZP basis set (cut-off radii for s-, p-, and d-orbitals are Rs = 9 Bohr, Rpd = 11 Bohr for Si) was used for STM image calculations [10]. Plane-wave cut-off for the grid is 200 Ry. Monkhorst-Pack k-point grids [11]: 2 × 2 × 1 for (1 1 0)-(5 × 8) and *c*(8 × 10), 1 × 4 × 1 for (1 1 0)-(16 × 2), and 2 × 4 × 1 for (17 15 1)-(2 × 1). Force tolerance: 0.01 eV/Å.
Description of data collection	The atomic structures reported here were obtained using the standard hydrogenated-slab first-principles density functional theory calculations. Calculated bulk Si lattice constant was 5.42 Å.
Data source location	Institution: A.V. Rzhanov Institute of Semiconductor Physics City/Town/Region: Novosibirsk Country: Russia
Data accessibility	With the article
Related research article	R.A. Zhachuk, A.A. Shklyaev Universal building block for (1 1 0)-family silicon and germanium surfaces Applied Surface Science 494 (2019) 46-50 https://doi.org/10.1016/j.apsusc.2019.07.144

**Table:** 

**Value of the Data** • Our data provide Si(1 1 0) and Si(17 15 1) surface atomistic models and related calculated STM images being in agreement with all available experimental STM data. The atomic coordinates can be directly reused in other calculations related to (1 1 0) surfaces of silicon and germanium. • Sharing our data is useful for the physicist and material scientists working with the Si(1 1 0) and Ge(1 1 0) surfaces. Experimentalists such as crystallographers and crystal growers will benefit from our surface structural models and calculated STM images. Computational physicists will take advantage from our datasets which are represented by atomistic coordinates. • The models allow to derive experimentally testable hypotheses and to interpret experimental data correctly. The calculated STM images will allow researchers to sustain further our models validation. In addition, the coordinates can be used in future first-principles calculations in conjunction with experimentalists working with Si(1 1 0) and Ge(1 1 0) surfaces.

## 1 Data

The dataset contains the raw relaxed atomic coordinates and related calculated STM images for various Si(110) and Si(17 15 1) surface atomic models. Atomic structure relaxations were performed using the SIESTA code [5], while the constant-current STM images were calculated using the WSxM software [9].

Table 1 lists the shared files with relaxed atomic coordinates of the following atomic models: the adatom-tetramer-interstitial (ATI) model of the Si(110)-(16 × 2) surface by Stekolnikov et al. [2], tetramer heptagonal- and tetragonal-ring stepped model (THTR) of the Si(110)-(16 × 2) surface by T. Yamasaki et al. [3] and the surface atomic models developed on the basis of the universal building block (UBB models) proposed in Ref. [1]: (110)-(16 × 2), (17 15 1)-(2 × 1), (110)-(5 × 8) and (110)-*c*(8 × 10). The coordinates in files are given in XYZ format (Å units). The atomic structures can be visualized using a number of freeware programs like: VESTA, GDIS, JMOL, MOLEKEL, VMD, RASMOL etc. The translation vectors for each surface structure are also given in Table 1.

**Table 1:** List of shared files with atomic coordinates with descriptions.

Surface	Model	Filename	Translation vectors (Å)			
Si(1 1 0)-(16 × 2)	ATI	Si(110)-16x2-ATI.xyz	V1	50.067815	2.212705	0.0
	THTR (pos.)	Si(110)-16x2-THTR+.xyz	V2	0.0	13.276234	0.0
	THTR (neg.)	Si(110)-16x2-THTR-.xyz	V3	0.0	0.0	30.0
	UBB	Si(110)-16x2-UBB.xyz				
Si(17 15 1)-(2 × 1)	UBB	Si(17151)-2x1-UBB.xyz	V1	25.107138	7.7445	0.0
			V2	0.0	13.276234	0.0
			V3	0.0	0.0	30.0
Si(1 1 0)-(5 × 8)	UBB	Si(110)-5x8-UBB.xyz	V1	–15.6462	22.1271	0.0
			V2	25.034	17.7017	0.0
			V3	0.0	0.0	30.0
Si(1 1 0)-*c*(8 × 10)	UBB	Si(110)-c(8 × 10)-UBB.xyz	V1	3.1292	–30.9779	0.0
			V2	28.1631	–13.2762	0.0
			V3	0.0	0.0	30.0

**Fig. 1:**
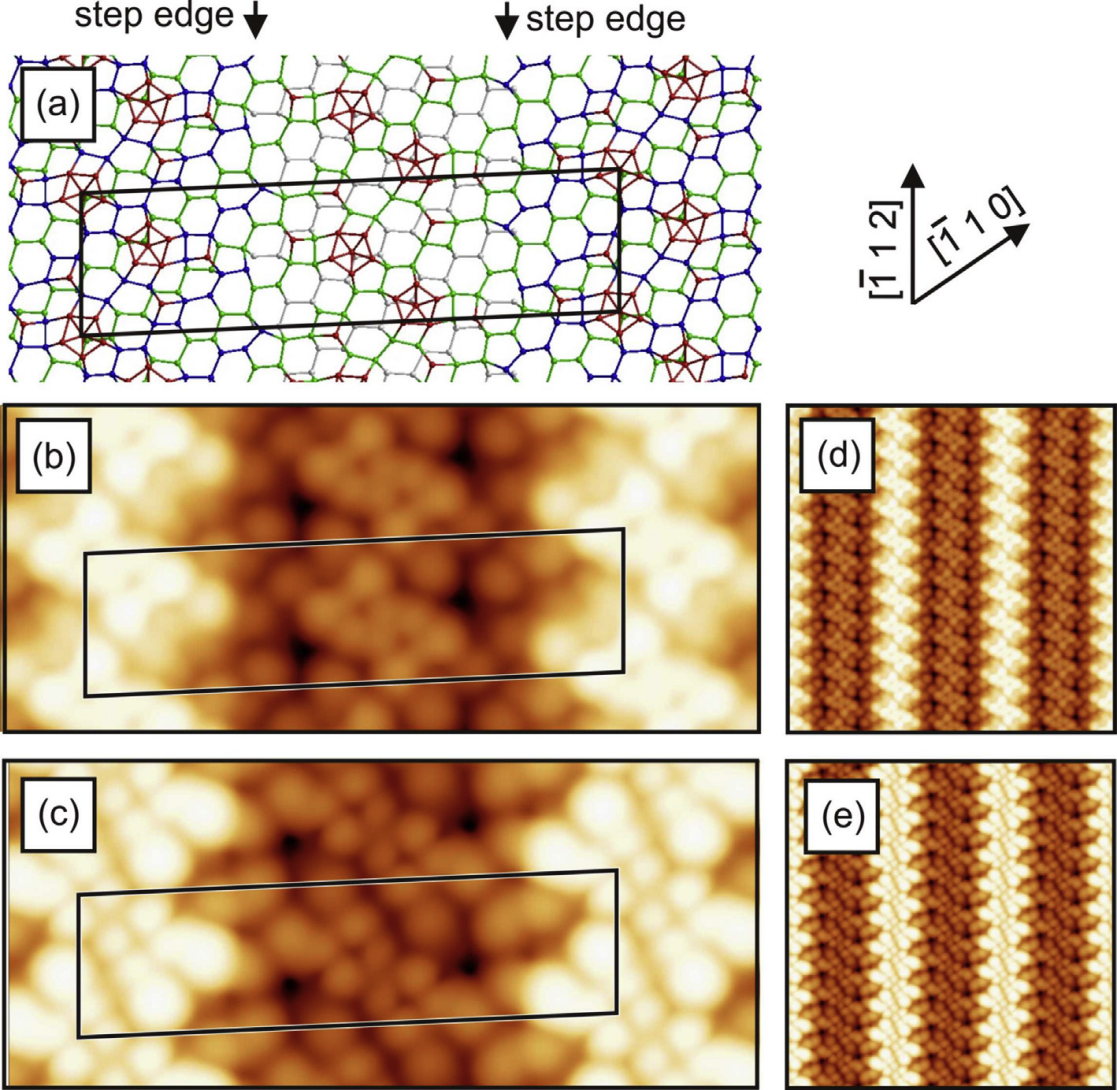
(a) ATI model of the Si(110)-(16 × 2) surface. The atoms of the first, second and third layers are marked in blue, green and white, respectively. Adatoms and pentamer atoms are marked in red. (b)–(e) Calculated constant-current STM images using the model shown in (a). (b), (d) *U* = +1.0 V. (c), (e) *U* = −1.0 V. The unit cell is outlined in (a), (b) and (c). The image size in (d), (e) is 150 × 150 Å^2^.

**Fig. 2:**
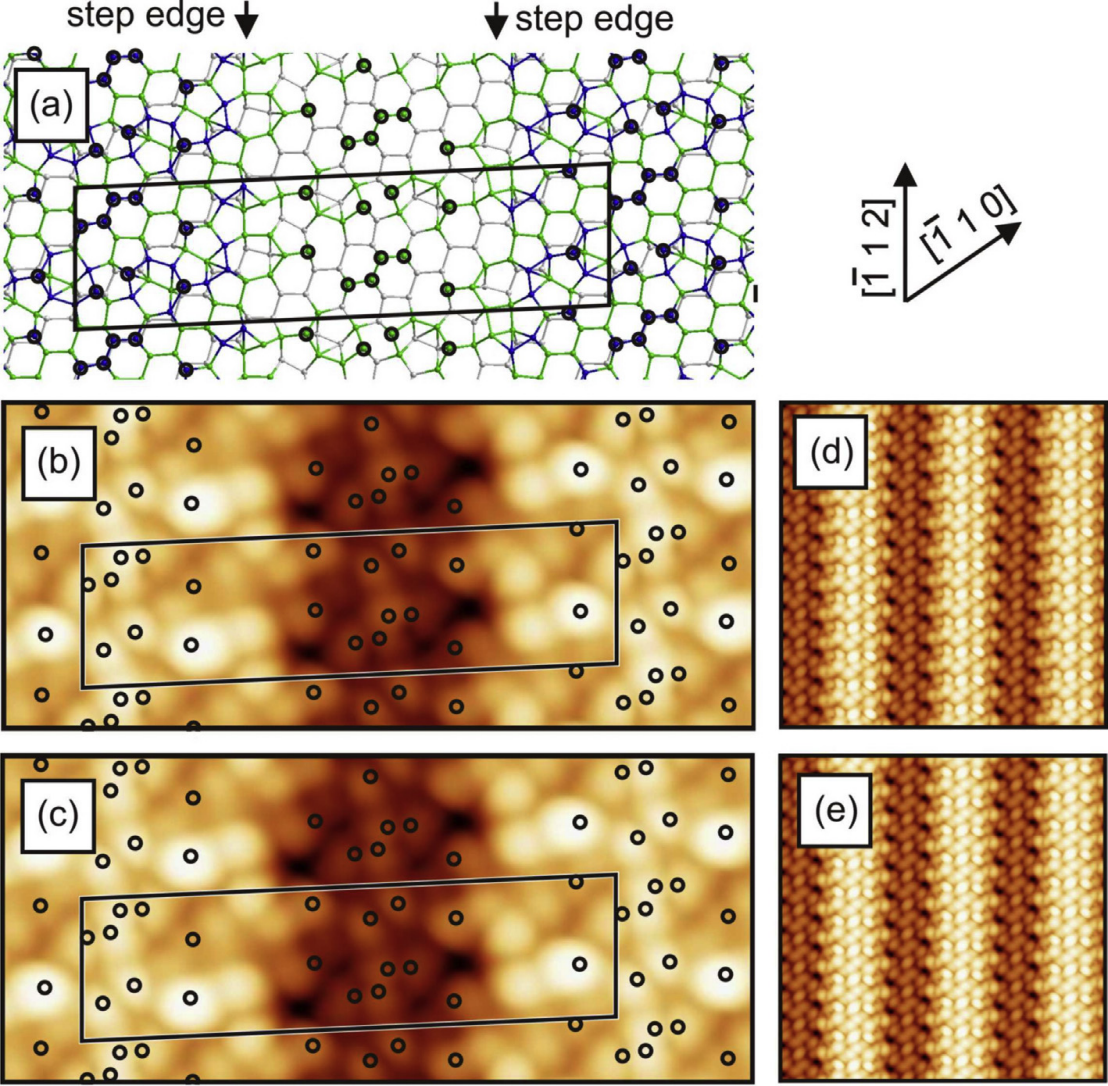
(a) THTR stepped model of the Si(110)-(16 × 2) surface (positive buckled configuration). The atoms of the first, second and third layers are marked in blue, green and white, respectively. The black circles indicate the positions P1–P5, where the bright spots in STM images are expected according to T. Yamasaki et al. [3]. (b)–(e) Calculated constant-current STM images using the THTR stepped model, *U* = +1.0 V. (b), (d) Positive buckled configuration. (c), (e) Averaged STM image using positive and negative buckled surface configurations. The unit cell is outlined in (a), (b) and (c). The Image size in (d), (e) is 150 × 150 Å^2^.

**Fig. 3:**
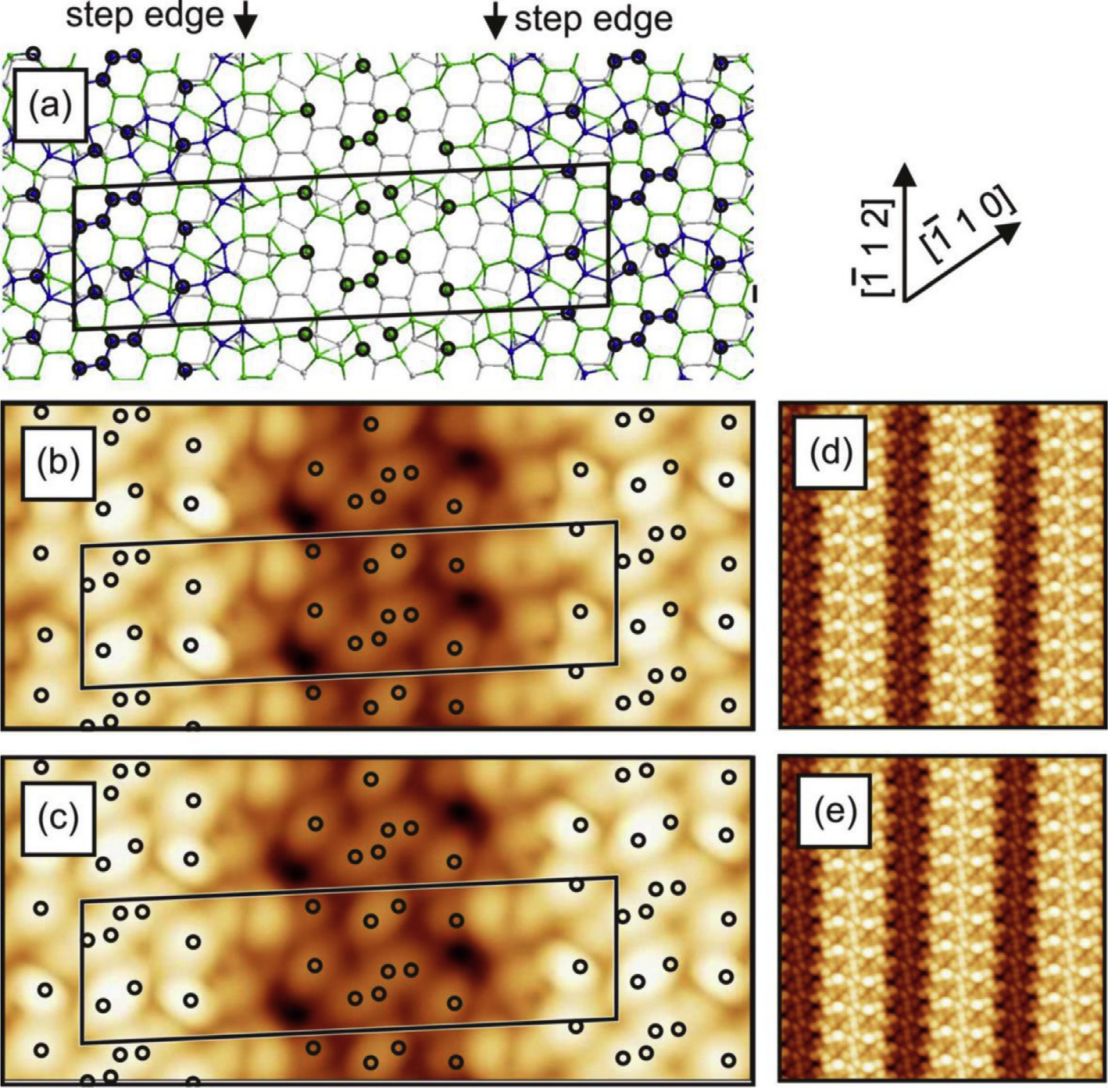
(a) THTR stepped model of the Si(110)-(16 × 2) surface (positive buckled configuration). The atoms of the first, second and third layers are marked in blue, green and white, respectively. The black circles indicate the positions P1–P5, where the bright spots in STM images are expected according to T. Yamasaki et al. [3]. (b)–(e) Calculated constant-current STM images using the THTR stepped model, *U* = −1.0 V. (b), (d) Positive buckled configuration. (c), (e) Averaged STM image using positive and negative buckled surface configurations. The unit cell is outlined in (a), (b) and (c). The image size in (d), (e) is 150 × 150 Å^2^.

**Fig. 4:**
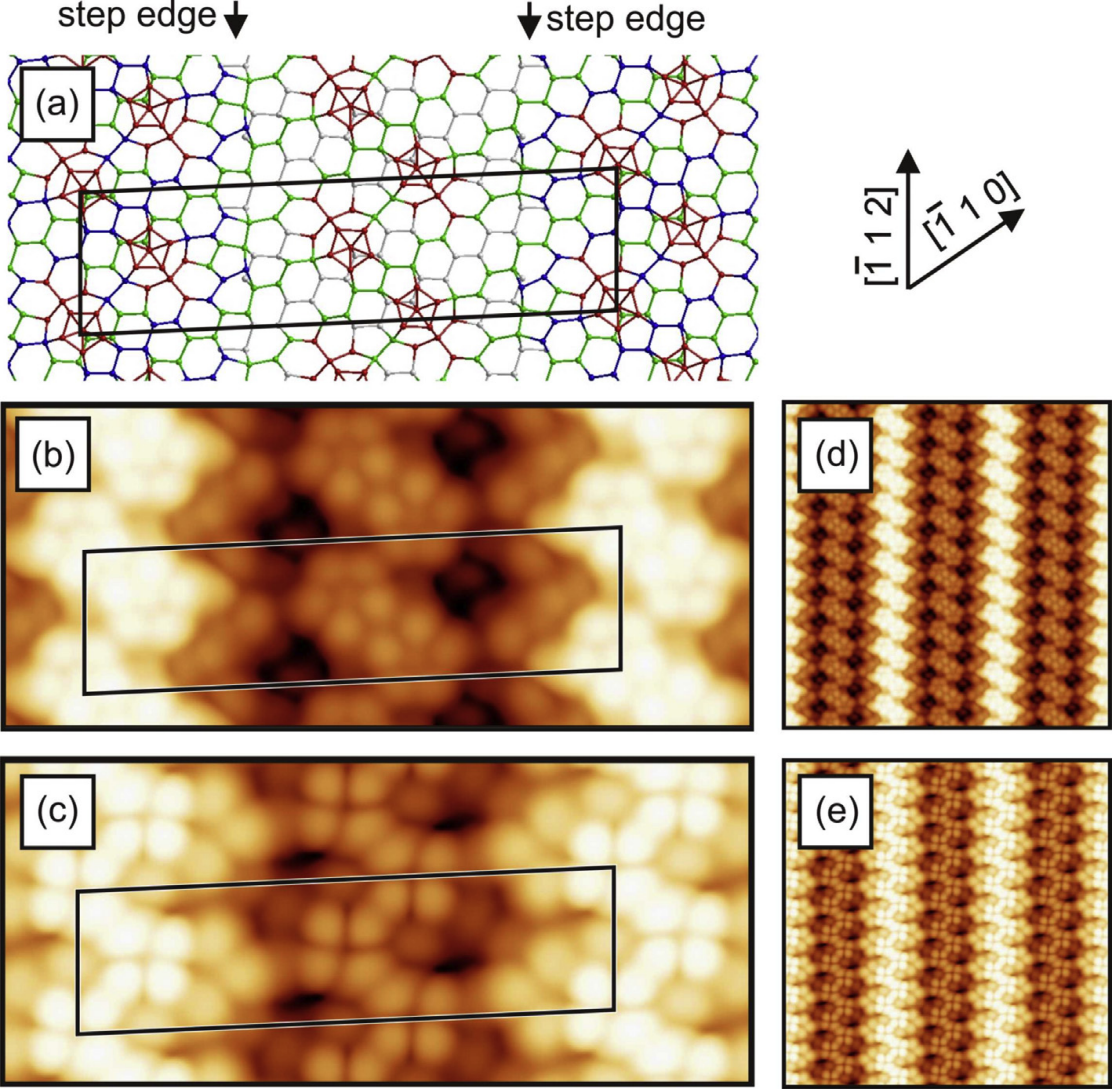
(a) UBB model of the (110)-(16 × 2) silicon and germanium surfaces. The atoms of the first, second and third layers are marked in blue, green and white, respectively. Additional atoms and atoms strongly shifted from their ideal (110) lattice positions are marked in red. (b)–(e) Calculated constant-current STM images using the model, shown in (a), and assuming a silicon surface. (b), (d) *U* = +1.0 V. (c), (e) *U* = −1.0 V. The unit cell is outlined in (a), (b) and (c). The image size in (d), (e) is 150 × 150 Å^2^.

**Fig. 5:**
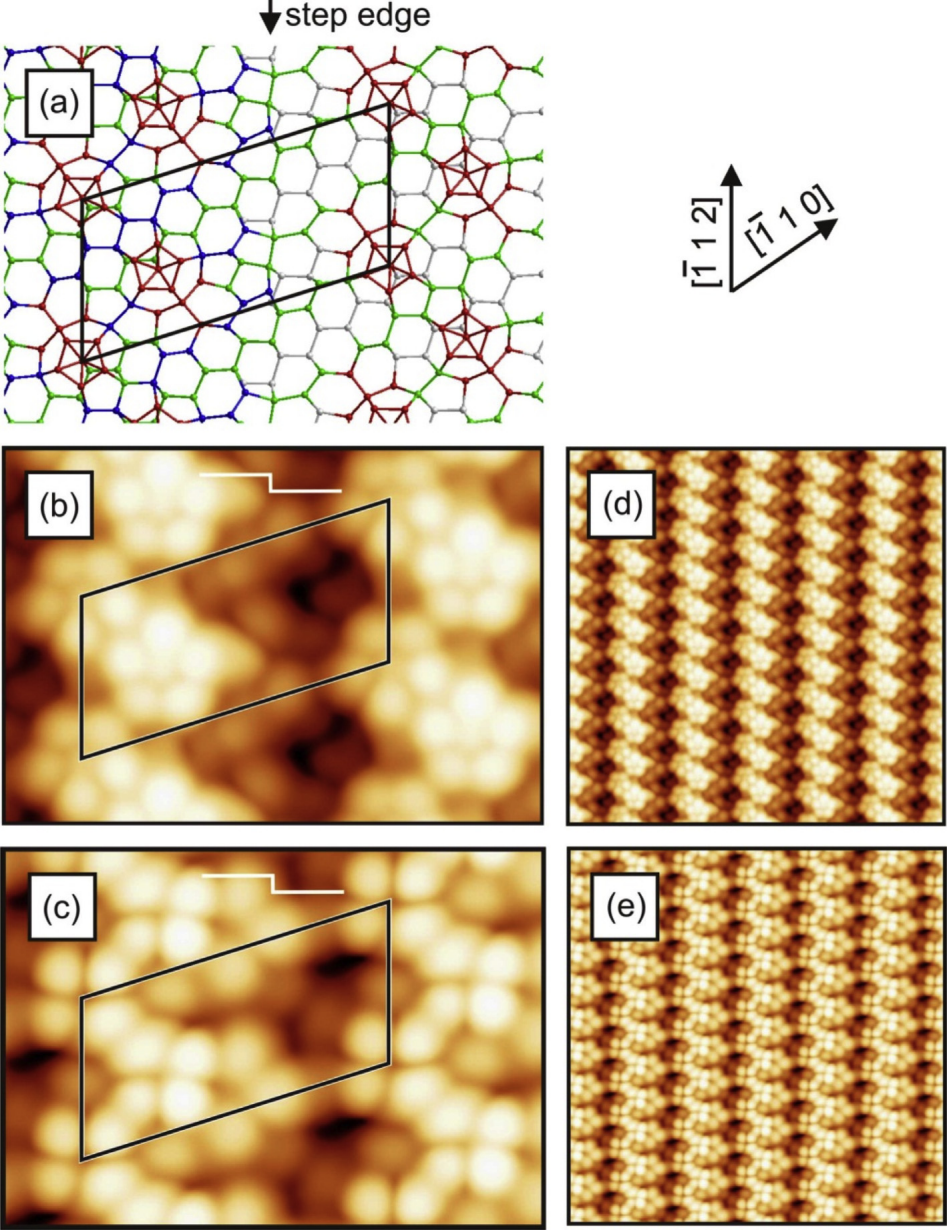
(a) UBB model of the (17 15 1)-(2 × 1) silicon and germanium surfaces. The atoms of the first, second and third (110) layers are marked in blue, green and white, respectively. Additional atoms and atoms strongly shifted from their ideal lattice positions are marked in red. (b)–(e) Calculated constant-current STM images using the model, shown in (a), and assuming a silicon surface. (b), (d) *U* = +1.0 V. (c), (e) *U* = −1.0 V. The unit cell is outlined in (a), (b) and (c). The image size in (d), (e) is 150 × 150 Å^2^.

**Fig. 6:**
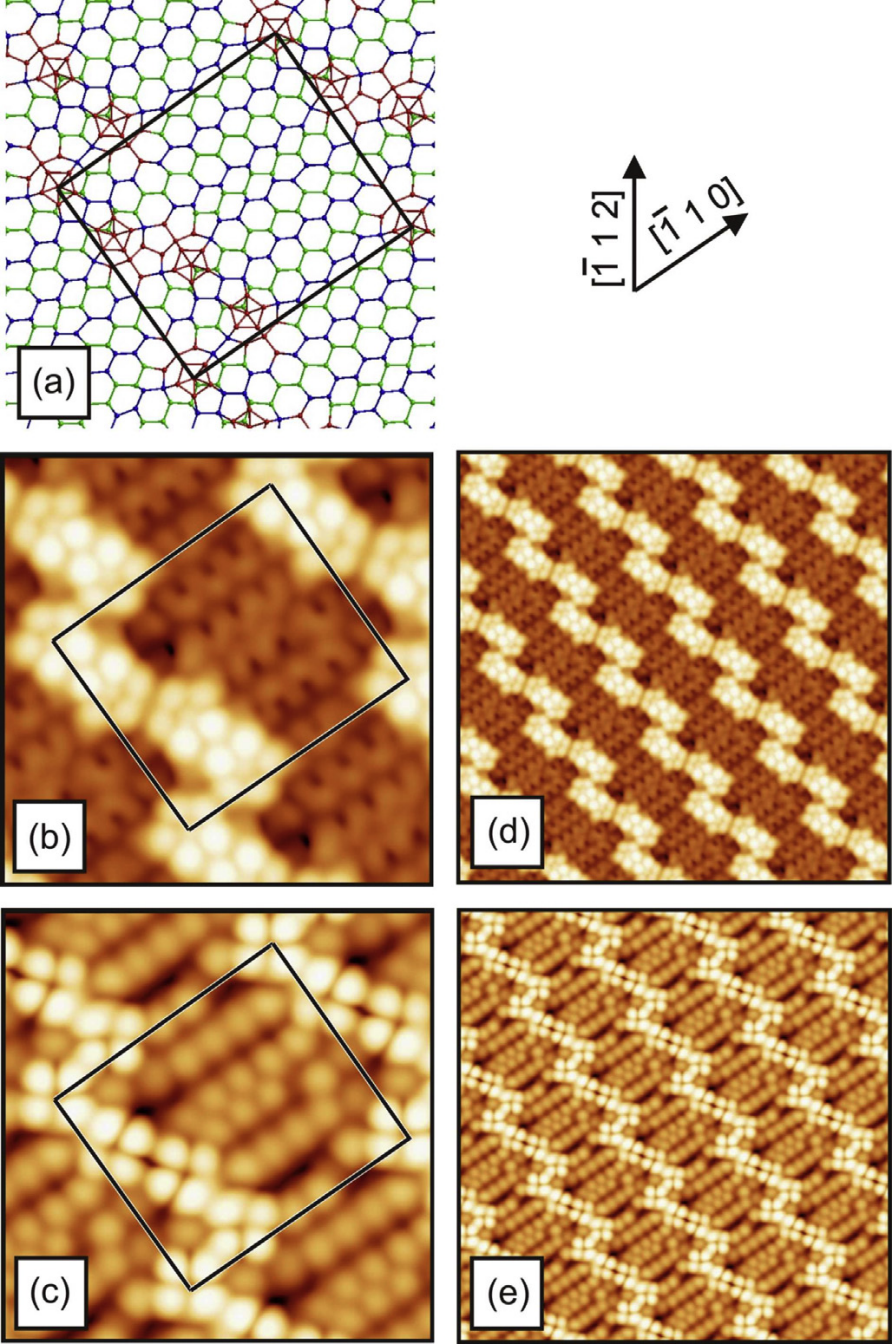
(a) UBB model of the (110)-(5 × 8) silicon and germanium surfaces. The atoms of the first and second (110) layers are marked in blue and green, respectively. Additional atoms and atoms strongly shifted from their ideal lattice positions are marked in red. (b)–(e) Calculated constant-current STM images using the model, shown in (a), and a assuming silicon surface. (b), (d) *U* = +1.0 V. (c), (e) *U* = −1.0 V. The unit cell is outlined in (a), (b) and (c). The image size in (d), (e) is 150 × 150 Å^2^.

**Fig. 7:**
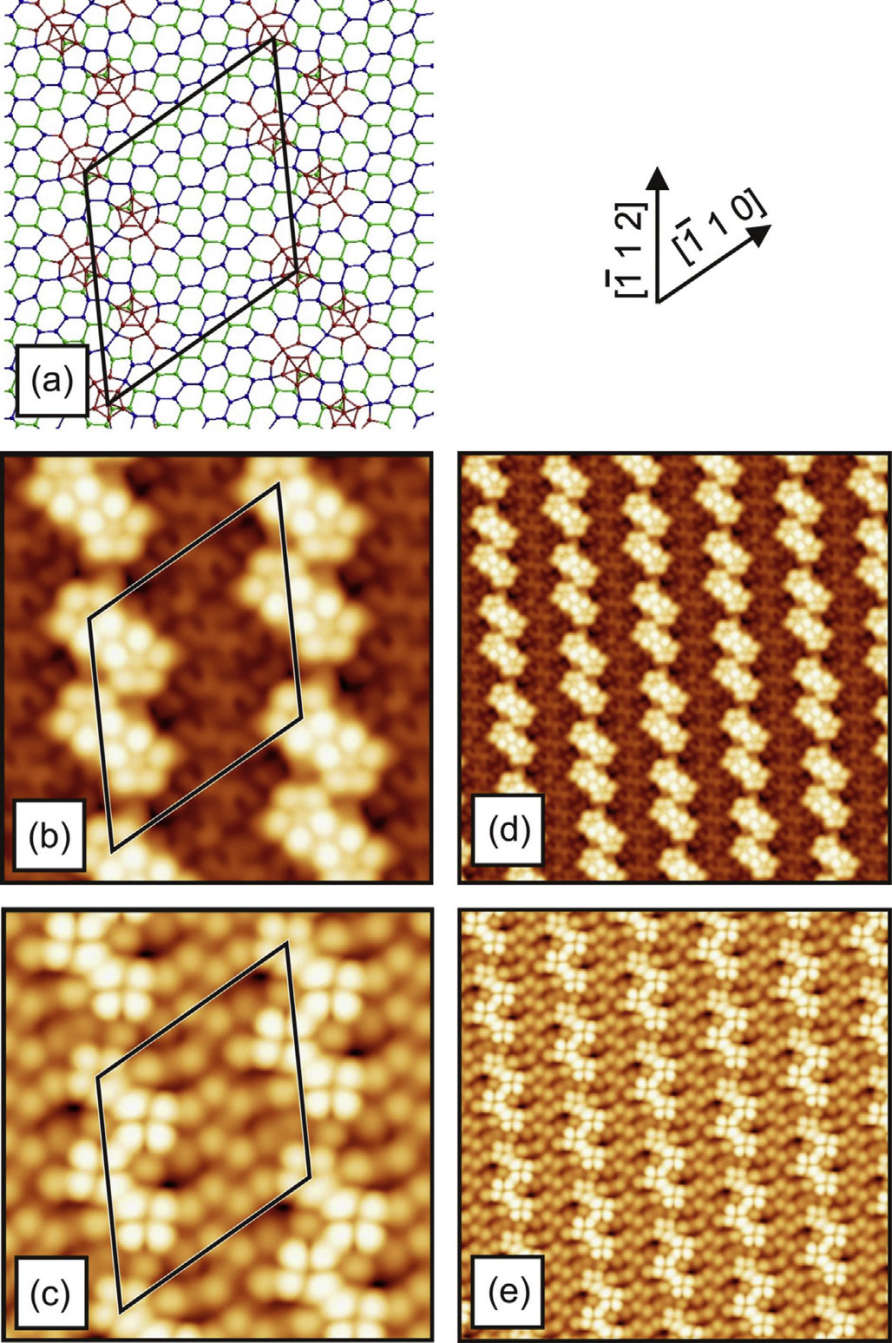
(a) UBB model of the (110)-*c*(8 × 10) silicon and germanium surfaces. The atoms of the first and second (110) layers are marked in blue and green, respectively. Additional atoms and atoms strongly shifted from their ideal lattice positions are marked in red. (b)–(e) Calculated constant-current STM images using the model, shown in (a), and assuming a silicon surface. (b), (d) *U* = +1.0 V. (c), (e) *U* = −1.0 V. The unit cell is outlined in (a), (b) and (c). The image size in (d), (e) is 150 × 150 Å^2^.

Fig. 1, Fig. 2, Fig. 3, Fig. 4 show three different models of the Si(110)-(16 × 2) surface and its related calculated bias-dependent STM images. Fig. 1 shows the ATI atomic model of the Si(110)-(16 × 2) surface by Stekolnikov et al. [2] (Fig. 1(a)) and its respective calculated constant-current STM images (Fig. 1(b)–(e)). The THTR stepped model of the Si(110)-(16 × 2) surface by T. Yamasaki et al. [3] (positive buckled configuration) and its respective calculated constant-current STM images are shown in Fig. 2, Fig. 3. Fig. 4 shows the UBB model of the Si(110)-(16 × 2) surface [1] (Fig. 4(a)) and its related calculated constant-current STM images (Fig. 4(b)–(e)). The UBB atomic models of the (17 15 1)-(2 × 1), (110)-(5 × 8), (110)-*c*(8 × 10) surfaces [1] and their calculated STM images are shown in Fig. 5, Fig. 6, Fig. 7 respectively.

## 2 Experimental design, materials, and methods

The structural models presented here are built upon a vast number of STM data obtained on (1 1 0) silicon and germanium surfaces and their vicinals published in the literature [see, for example [12,13]].

The work is performed using first-principles calculations. The calculations were carried out using the pseudopotential [4] density functional theory SIESTA code [5] within the local density approximation to the exchange and correlation interactions between electrons [6]. The valence states were expressed as linear combinations of the Sankey-Niklewski-type numerical atomic orbitals [5]. In the present calculations, the polarized double-ζ functions (DZP) were assigned for all species. This means two sets of s- and p-orbitals plus one set of d-orbitals on silicon atoms, and two sets of s-orbitals plus a set of p-orbitals on hydrogen atoms. The electron density and potential terms were calculated on a real space grid with the spacing equivalent to a plane-wave cut-off of 200 Ry. The calculations were performed using 6 layers thick slabs (7 layer slabs for the (16 × 2) reconstruction) terminated by hydrogen from one side. A 18 Å thick vacuum layer was used. We used specific k-point grids for each surface reconstruction/slab, depending on its respective lateral dimensions, namely: 2 × 2 × 1 for (1 1 0)-(5 × 8) and *c*(8 × 10), 1 × 4 × 1 for (1 1 0)-(16 × 2), and 2 × 4 × 1 for (17 15 1)-(2 × 1) [11]. The positions of all slab atoms (except for the Si atoms in two layers at the bottom and all H atoms) were fully optimized until the atomic forces became less than 0.01 eV/Å. In the bulk case, our calculation yields a cubic lattice constant of Si *a*_0_ = 5.420 Å.

The geometry optimizations were performed using the standard bulk-optimized DZP basis set, with energy shift parameter set to 100 meV and split norm set to 0.25. The constant-current STM images were produced based on the Tersoff-Hamann approximation [7] using the eigenvalues and eigenfunctions of the Kohn-Sham equation [8] for a relaxed atomic structure. For this purpose we performed additional SCF-calculation using the relaxed atomic structure and the surface optimized basis set (cut-off radii for s-, p-, and d-orbitals are Rs = 9 Bohr, Rpd = 11 Bohr for Si) [10]. The resulting local density of electronic states (LDOS) files were used to produce the calculated STM images in the WSxM software [9].
